# 

**Published:** 1895-12-28

**Authors:** 


					TME HOSPITAL, ^-'^BSOV UTELY PU RE, therefore BEST. December 28th, 1895.
CADBURYS ^ CADBURY'S
> COCOA JO m COCOA
"The standard of highest purity at present NO ALKALIES USED.
attainable."?Laneet. *
'RtT/CH HOSPl.TAL
No. 483. Vol. XIX.
Saturday,
Dec. 28th, 1895.
WITH EXTRA NURSING SUPPLEMENT. "ffig-F7B2S3*
Registered at the General Post Office as a Newspaper for Monthly Parts, 6cL; post free, 8d.
transmission in the United Kingdom and Abroad.] Ditto per Annum, 8s.6cL, post free.

				

